# Medicine in Mandarin: Introducing Native Language Training in a Medical School Curriculum

**DOI:** 10.15694/mep.2019.000205.1

**Published:** 2019-11-15

**Authors:** Yingchao Zhong, Elaine A. Liu, Kelly Z. Young, Jane Miller, Michael Heung

**Affiliations:** 1 University of Michigan Medical School; 2 Michigan Medicine

**Keywords:** limited English proficiency, language barriers, Mandarin, medical interpreter, cultural competency, cross-cultural communication

## Abstract

This article was migrated. The article was marked as recommended.

**Background:**Chinese Americans are one of the fastest growing populations in the United States. However, 41% of Chinese Americans are not English proficient.

**Methods:**In 2014, Medicine in Mandarin was established by the University of Michigan Medical School (UMMS) as a pre-clinical elective taught by a nationally certified healthcare Mandarin interpreter. A 32-hour curriculum was developed, including both didactics and interactive elements. Assessments included quizzes, standardized patient interviews, and a final exam. An evaluation was administered upon course completion, and a post-course survey was administered to graduates at least six months after course completion.

**Results:**Between 2014 and 2017, the elective graduated 25 students, of whom 23 completed the course evaluation and 22 completed the post-course survey. Prior to the course, 9% of students felt comfortable practicing medicine in Mandarin. This increased to 78% of students post-course. When asked about subsequent clinical experiences, 82% of students reported having applied medical Mandarin skills. Overall, 96% rated the course as very good or excellent.

**Conclusion:**A Medicine in Mandarin elective was well-received by students and improved their comfort in providing medical care in Mandarin. Additional study is warranted to examine the potential clinical impact of this course.

## Introduction

People of Chinese descent comprise a substantial and rapidly growing proportion of the US population and represent the largest Asian American ethnic group (Hoeffel *et al.*, no date). However, 41% of all Chinese Americans are not English proficient, indicating that many Chinese-American patients rely on language assistance in health care settings ( Mitchell, 2017). Limited English proficiency (LEP) patients encounter challenges in the healthcare system, from strained communication with healthcare providers to a lack of utilization of the healthcare process ( Gulati *et al.*, 2012). LEP patients are more likely to experience adverse medication reactions, not adhere to prescribed treatments, require longer emergency care visits, receive fewer recommended services, and be less satisfied with their care ( Moreno, Walker and Grumbach, 2010). Indeed, for the LEP immigrant, navigating the healthcare system is a challenging and formidable task.

As the correlation between decreased language congruence, cultural competence, and patient involvement and satisfaction has been documented ( Gulati *et al.*, 2012), one way of mitigating these issues is by narrowing the patient-provider language gap by using interpreters. The Joint Commission states that “hospitals must develop a system to provide language services to address the communication needs of patients whose preferred language is not English” ( *The Joint Commission: Advancing Effective Communication, Cultural Competence, and Patient- and Family-Centered Care: A Roadmap for Hospitals*, 2010). Professional interpreters are the gold-standard, providing a significantly lower proportion of interpretation errors ( Flores *et al.*, 2012). However, the use of professional services is often costly, limited, administratively difficult, and lacking in standardization ( Hornberger, Itakura and Wilson, 1997; Regenstein *et al.*, 2008). As a result, healthcare teams often resort to using ad hoc interpreters based on convenience, the use of which has been documented to be error-prone ( Flores *et al.*, 2012; Vela, Fritz and Jacobs, 2015). Even in well-resourced environments, professional interpreter services are a limited resource, and utilization is not highly correlated with demand ( Hornberger, Itakura and Wilson, 1997).

Training physicians to provide care in non-English languages is another approach that could complement the use of interpreters to provide better care for LEP patients. At the University of Michigan Medical School (UMMS), a Medicine in Mandarin (MiM) course was developed in response to student interest and demand, the growing Mandarin speaking patient population, and the need for more physicians who can effectively communicate in Mandarin and provide culturally competent care. Here, we discuss the development and implementation of MiM. We also discuss program evaluations, curriculum changes, and lessons learned that have shaped the course over the first three years.

## Methods

A nationally certified healthcare Mandarin interpreter/trainer was recruited as course instructor, and a faculty physician was invited to provide clinical expertise and oversee the administrative aspects of the course. A curriculum committee, consisting of the course instructor, faculty physician, and four student leaders was formed to draft the course proposal, which included the course description, learning objectives, required learning experiences, assessments and grading, student and program evaluation, and institutional objectives.

Because the endeavor of learning Mandarin was considered out of the scope of this course, enrollment criteria required a conversational level of Mandarin fluency. Despite conversational fluency, few Mandarin speaking students were familiar with medical terminology and clinical-level communication in Mandarin. Furthermore, the ability to read and write Chinese was not an inclusion criterion, as the focus was on verbal communication. Therefore, all written course materials were presented with pinyin, the Romanized pronunciation of Mandarin. Interested students completed a two-part application consisting of a) an oral recording of the student speaking Mandarin to assess fluency, and b) an essay regarding why they were interested in the course and how they hoped to apply the skills learned. Applications were reviewed and students selected by the course curriculum committee. Although the majority of students had Chinese heritage, 5 out of the 25 (20% of students) did not, but had studied Chinese as a foreign language.

### MiM Curriculum

A 32-hour curriculum (16 weeks, two hours per week) was developed for students to achieve mastery of a number of course objectives, including performing a medical history and general physical in Mandarin, participating in health counseling conversations in Mandarin, and understanding important Chinese cultural beliefs that may influence health care decision-making ( Table 1). Course content was adapted from the medical school curriculum and designed to temporally parallel and complement information taught in the medical school curriculum. This allowed students to learn content-specific Mandarin terminology shortly following its introduction in English. As the emphasis of MiM was on clinical utility, the course focused on terms and phrases a provider is likely to use in conversations with patients.

**Table 1.** Curriculum Overview, Learning Objectives, and Teaching Methods

Curriculum Overview	Schedule	Learning Objectives	Teaching Methods
**Vocabulary**			
Basic Common Phrases	Week 1	Incorporate foundational medical Mandarin vocabulary, phrases and terminology into conversation.	Lecture Role Play Scenarios Vocabulary Games
General Terminology and Body Parts	Week 2		
Cardiovascular and Respiratory System	Week 5		
Musculoskeletal System	Week 6		
Central Nervous System	Week 7		
Endocrine System	Week 8		
Reproductive System	Week 9		
Gastrointestinal System	Week 10		
Renal System	Week 11		
**Sociocultural Topics**			
Chinese and Chinese American Culture, Sociocultural Aspects, and Health Beliefs	Week 3	Describe Chinese cultural and socio-cultural beliefs that may influence healthcare decisions, including Chinese nutrition and lifestyle, common illnesses within the Chinese population, and views on end of life care.	Guest LectureGroup Discussion
Common Illnesses in the Chinese Community	Week 13		Group Presentations
Patient Decision Making (End of Life Care, Palliative Care, Hospice, and Advance Directives)	Week 14		LectureGroup Discussion
Chinese Diet, Nutrition, Lifestyle, and Exercise	Week 14		LectureGroup Discussion
**Clinical Application of Vocabulary**			
Introduction to the History and Physical Exam	Week 4	Conduct an H&P in Mandarin by example by observing a physician H&P.	Guest Lecture
History Intake and Physical Practice Session	Week 12	Interact with native Chinese speakers and practice conducting authentic H&Ps	Practice with Native Chinese Speakers
History Intake and Physical	Week 15	Perform an H&P and receive feedback on communication and clinical skills.	Standardized Patient Interaction

Each week, students learned and practiced medical vocabulary related to an organ system using didactic lectures. Active learning techniques (group discussions, role playing, games, and peer presentations) ( Table 1) facilitated students’ use of new vocabulary in conversation. Students reinforced vocabulary through an online resource that featured terms alongside their Chinese characters, pinyin, and audio recordings with proper pronunciation.

To help prepare learners to navigate complex cultural and sociological issues, the course introduced students to Chinese cultural and sociocultural topics, varying from health beliefs, Chinese nutrition and lifestyle, to common illnesses in the Chinese community and approaches to end of life care. This material was presented through guest lectures led by physicians and other healthcare providers, who shared their experiences handling relevant sociocultural issues and providing culturally-sensitive care.

History and physical exam (H&P) training was conducted by a physician who practices in Mandarin, and included a demonstration of an H&P in Mandarin, followed by a session where students practiced these skills through role-playing. Students then practiced performing H&Ps on Mandarin-speaking community volunteers with chronic ailments at a local senior community center, under the supervision of the course instructor. This provided an opportunity for students to interact with native Chinese speakers and practice taking authentic, unscripted H&Ps.

This curriculum was approved by the UMMS Curriculum Policy Committee as a formal elective with inclusion on medical school transcripts.

### Student Assessment

Assessments included weekly multiple choice quizzes, a video-recorded H&P with a standardized patient, and a final multiple choice exam. Weekly quizzes, consisting of roughly ten multiple choice questions based on vocabulary introduced earlier that week, were designed to encourage engagement with new material and provide frequent feedback. An example of a multiple choice question is an audio recording of an anatomical term in Mandarin, asking the student to select the corresponding term in English. The final exam consisted of fifty multiple choice questions that comprehensively tested vocabulary presented throughout the course. It was given in a similar format to weekly quizzes and graded by percent correct. All assessments were delivered using vocal recordings and/or pinyin to assess listening comprehension. Finally, the instructor assessed students’ level of engagement as evidenced by in-class participation.

To assess students’ abilities to perform an H&P, a culturally appropriate scenario was developed and community members fluent in Mandarin were recruited and trained to be standardized patients (SPs). This exercise was done twice, first approximately midway through the course, and again at the end of the course. The SP interview was done with the same scenario and standardized patient both times for consistency. The SP case and assessment checklist were adapted directly from existing standardized medical school SP experiences, but patient features were altered to make the case culturally relevant to native Mandarin speakers (for example, the patient takes a ginseng supplement). Students were assessed for proficiency in language delivery and fluency across 22 checklist areas. Example areas include “Introduces himself/herself by name and position”, and “Asks about patient’s diet”. Standardized patients provided direct feedback immediately following the interview, and the instructor also assessed the interviews through video recordings.

### Course Evaluation

Immediately following completion of the elective, students completed course evaluations to determine course effectiveness. Questions asked students about their comfort level practicing medicine in Mandarin before and after taking the elective, using vocabulary, taking H&Ps, and understanding sociocultural issues ( Table 2). Students were also asked to submit anonymous feedback on course strengths and areas for improvement.

After MiM course graduates entered the clinical phase of their medical school training, they were asked to complete a post-course survey to evaluate clinical effectiveness of the elective. Students were surveyed for frequency of encounters with Mandarin speaking patients, frequency of application of skills taught in the elective, and areas in which taking the elective helped their interaction with Mandarin speaking patients. Finally, students were asked to provide narrative comments of how the elective impacted their patient care experiences.

All data were de-identified or collected anonymously. All aspects of this project were reviewed by the UMMS Institutional Review Board and were determined to be exempt from full review because this study did not meet the definition of human subjects research (i.e. the course activities were the object of study, and not the students).

## Results/Analysis

Twenty-five pre-clinical students completed the MiM elective from 2014 to 2017 (N=6 2015, N=10 2016, N=9 2017).

In terms of student assessment, on average, students performed 85.9% of the tasks proficiently during their mid-way SP experience, as determined by the SP. In comparison, students showed an improvement during the end of term SP interview, and performed on average 92.8% of the tasks proficiently (paired T-test p=0.027).

Twenty-three of 25 (92%) students completed the post-course evaluation. Prior to taking the elective, only two (8.7%) students agreed that they felt comfortable practicing MiM, while 18 (78.3%) students disagreed. Following completion of the elective, 18 (78.3%) students agreed that they felt comfortable practicing MiM and no (0%) students disagreed ( Table 2). All 23 (100%) students agreed that the elective improved their ability to take a history, and 20 (87%) students agreed that the elective improved their ability to perform a physical exam in Mandarin ( Table 2).

**Table 2.** Course Evaluation Results

Evaluation Question	Strongly Agree or Agree, N (%)	Neutral, N (%)	Strongly Disagree or Disagree, N (%)
Prior to this elective, I was comfortable with my ability to practice medicine in Mandarin (N=23)	2 (8.7%)	3 (13%)	18 (78.3%)
After completing this elective, I am comfortable with my ability to practice medicine in Mandarin. (N=23)	18 (78.3%)	5 (21.7%)	0 (0%)
This elective improved my ability to use Mandarin anatomical and medical terminology in a clinical setting. (N=23)	22 (95.7%)	1 (4.3%)	0 (0%)
This elective improved my ability to take a history in Mandarin. (N=23)	23 (100%)	0 (0%)	0 (0%)
This elective improved my ability to perform a physical using Mandarin. (N=23)	20 (87%)	2 (8.7%)	1 (4.3%)
This elective improved my understanding of sociocultural characteristics of Chinese communities.(N=23)	22 (95.7%)	1 (4.3%)	0 (0%)
This elective improved my understanding of how to provide health care to members of Chinese communities. (N=22)	20 (90.9%)	2 (9.1%)	0 (0%)
Overall, the twice-weekly course sessions improved my ability to practice medicine in Mandarin. (N=23)	23 (100%)	0 (0%)	0 (0%)
Practicing patient histories in Mandarin with other students improved my ability to practice medicine in Mandarin. (N=23)	20 (87%)	2 (8.7%)	1 (4.3%)
Practicing patient interviews in Mandarin with Standardized Patient instructors improved my ability to practice medicine in Mandarin. (N=23)	22 (95.7%)	1 (4.3%)	0 (0%)
Practicing patient interview and physical exam with volunteers at Parkway Meadows Senior Apartment improved my ability to communicate with patients in Mandarin (N=17)	15 (88.2%)	2 (11.8%)	0 (0%)
The guest speaker experiences enhanced my learning experience. (N=23)	19 (82.6%)	4 (17.4%)	0 (0%)
The interpreter-shadowing experience at UMHS improved my ability to practice medicine in Mandarin. (N=6)	6 (100%)	0 (0%)	0 (0%)
The interpreter-shadowing experience at UMHS improved my ability to work with an interpreter. (N=6)	6 (100%)	0 (0%)	0 (0%)
The service-learning volunteer opportunity (UAAMSA Health Fair) enhanced my learning experience and improved my ability to practice medicine in Mandarin. (N=23)	20 (87%)	3 (13%)	0 (0%)
The course contributions of the Elective Instructor improved my ability to practice medicine in Mandarin. (N=23)	23 (100%)	0 (0%)	0 (0%)
The elective assessments (exams) provided an accurate indication of my knowledge. (N=23)	20 (87%)	3 (13%)	0 (0%)
**Evaluation Question**	**Excellent or Very Good**	**Good**	**Fair or Poor**
Overall, the elective was ...	22 (95.7%)	1 (4.3%)	0 (0%)

Teaching methods were designed to be interactive and practice-driven. Twenty (87%) students agreed that practicing patient histories with other students improved their abilities to practice medicine in Mandarin. Twenty-two (95.7%) students agreed that SPs improved their abilities to practice medicine in Mandarin. Furthermore, a unique aspect of the course was having a certified medical interpreter serve as the course instructor. All (100%) students agreed that the instructor improved their abilities to practice medicine in Mandarin ( Table 2).

Responders to the post-course survey included course alumni in various stages of clinical training, from clinical students to first-year residents. Twenty-two (88%) course alumni completed this survey (N=6 2015, N=9 2016, N=7 2017). Fourteen (63.6%) students reported encountering patients whose preferred language for healthcare is Mandarin at least once a year, and five (22.7%) students reported at least once a month. When asked how often students use the skills learned in MiM, 15 (68.2%) students reported at least once a year with seven (31.8%) students reporting at least once a month. Overall, the vast majority (N=20, 90.9%) of students agreed that MiM helped prepare them to communicate with Mandarin-speaking patients ( Table 3). Sixteen (72.7%) students agreed that communicating in the native language of the patient allowed them to provide better patient care, and all students agreed that medical language courses are an important part of a medical curriculum ( Table 3).

**Table 3.** Post-Course Survey Results

Survey Question	At least once a year, N (%)	At least once a month, N (%)	Less than once a year, N (%)
How often do you have a patient whose preferred language for healthcare is Mandarin? (N=22)	9 (40.9)	5 (22.7)	3 (13.6)
How often do you use the skills you learned in medicine in Mandarin? (N=22)	8 (36.4)	7 (31.8)	4 (18.2)
**Survey Question**	**Strongly agree or agree, N (%)**	**Neutral, N (%)**	**Strongly disagree or disagree, N (%)**
The Medicine in Mandarin course prepared me to communicate with Mandarin speaking patients (N=22)	20 (90.9)	1 (4.5)	0 (0)
The Medicine in Mandarin course provided me with valuable skills that I have applied in the clinical setting (N=22)	18 (81.9)	3 (13.6)	1 (4.5)
The Medicine in Mandarin course helped me understand cultural concerns of Chinese heritage patients (N=22)	20 (90.9)	2 (9.1)	0 (0)
Communicating in the native language of the patient allowed me to provide better patient care (N=22)	16 (72.7)	3 (13.6)	0 (0)
I believe medical language courses are an important part of a medical curriculum (N=22)	22 (100)	0 (0)	0 (0)
**Do you have a personal story of how the Medicine in Mandarin course impacted your patient care experience while on the wards? If so, please share below.**			
“When I...jumped in and started talking in Chinese, the mother’s face was quickly replaced with relief as she found she could communicate her wishes.”			
“Because I can communicate with Chinese patients, I’ve been able to quickly gain rapport and make patients feel comfortable enough to openly share their concerns and questions.”			
“Using my Medical Mandarin vocabulary, I explained pros and cons of different labor interventions, gave entire neonate discharge talks, and provided reassurance to patients’ many medical questions. I would not have felt prepared to do this without first having completed the Medical Mandarin elective.”			
“There was a Mandarin speaking mother with prolonged labor... She was very frightened, but I did my best to explain what was happening in Chinese. Without me, she would have had no idea what was happening.”			
“Being able to speak Chinese was immensely helpful...to make sure we understood what the patient’s wishes were and to evaluate how he was feeling to ensure that his pain and symptoms were sufficiently managed.”			
“During my intern year, there was a visiting scholar from China...he found it difficult to communicate with other physicians. After I translated for him, he was finally able to understand the procedures and criteria we use for the operations we do.”			
“There was no Mandarin speaker available, either in-house or by phone... I volunteered to speak to the family and was available afterwards for the family’s questions. Medical Mandarin gives us the tools to speak and care effectively for our patients.”			
“I am lucky to have been enrolled in the Medicine in Mandarin course as it really sharpened my medical Chinese knowledge and made me comfortable and confident in interviewing patients in Beijing [for my research project on hepatitis stigma]. Over the course of the summer, I was able to converse with hundreds of patients about their experiences living with a chronic disease.”			

## Discussion

In this study, we describe the development and early experience with a medical school language course designed to enhance clinical communication in Mandarin. We found that students perceived that the course was effective at preparing them for clinical interactions with Mandarin speaking patients. Virtually all students reported improved comfort in practicing medicine in Mandarin. This was true not only immediately following the course, but also as students began working with patients in the clinical setting.

To better care for LEP patients, many healthcare systems and hospitals have adopted in-person and phone interpreter services. Though interpreters have successfully improved integration of LEP patients into the healthcare system, the use of interpreters is costly and often limited to well-resourced healthcare centers ( Hornberger, Itakura and Wilson, 1997). In one national survey examining 71 high-capacity hospitals, only 40 (53.5%) hospitals provided in-person interpreting services for at least five languages, with varying degrees of reliance on telephonic interpretation services ( Regenstein *et al.*, 2008). Furthermore, the use of interpreters is also limited by interpreter availability and scheduling difficulties. Reliance on interpreter availability can result in long waits ( Hornberger, Itakura and Wilson, 1997) and may not be feasible for urgent situations. Therefore, even in well-resourced medical centers with adequate interpreter infrastructure, there remains a need for healthcare providers who can deliver care in a patient’s native language. MiM was developed to train such providers and succeeded at improving clinical skills in Mandarin, both based on students’ self-report and by the evaluation of a professional Mandarin interpreter.

Importantly, MiM graduates are not a replacement for trained interpreters. Rather, we believe that adoption of language electives in medical school curricula should complement the work of medical interpreters in clinical settings by increasing linguistic diversity and capacity, and thereby improve the overall patient experience for LEP patients. Reliance on interpreters can lead to impersonal physician-patient interactions and lengthen patient visits ( Fagan *et al.*, 2003), and multiple authors have advocated for multicultural medical education and more linguistic diversity among practicing physicians ( Tervalon and Murray-García, 1998; Moreno, Walker and Grumbach, 2010). A physician who is able to conduct parts of the clinical encounter directly with a patient, while having the self-awareness to request professional interpretation for more technically challenging situations, can step in when an interpreter is not readily available. Communicating in a patient’s native language may allow for better physician-patient rapport, promote a more trusting relationship, and increase the patient’s willingness to share important information; these themes were reflected in the testimonials provided by student graduates of MiM ( Table 3). Even when an interpreter is present, having native language training can help clinicians better understand the communication nuances that may be lost through interpretation.

To date, several medical schools (including New York University, Robert Wood Johnson, Harvard, Stanford, and University of North Carolina) have developed medical Mandarin elective courses for medical students. A comparison of online descriptions reveals diverse approaches to content delivery that vary in administrative support, instructor credentials, and course structure, with no existing model of consensus. Existing literature contains multiple examples of medical Spanish curricula across medical and other health professional schools ( Prince and Nelson, 1995; Bloom, Timmerman and Sands, 2006; Reuland *et al.*, 2008; VanTyle *et al.*, 2011). There has even been a national survey done on medical Spanish curricula in the United States ( Morales *et al.*, 2015). Although lessons can be learned from medical Spanish programs, each cultural group has specific sociocultural beliefs that influence their interaction and acceptance of the practice of medicine ( Jung *et al.*, 2017). However, besides a publication on a two-hour medical Mandarin workshop ( Zhang *et al.*, 2009), the authors are not aware of other formal reports describing curriculum development for medical Mandarin. Therefore, our study provides the first formal description of a MiM elective that can serve as a model for continued implementation at UMMS and future implementation at other medical schools around the nation.

In evaluating the effectiveness of the MiM elective, we relied heavily on student self-reports. Though we have shown strong evidence from the students’ perspective that this course was effective at teaching Medicine in Mandarin, we hope to confirm these findings with clinical-based evidence. To address this, future evaluations of the MiM elective will include patient survey assessments to elicit feedback from clinical encounters. We hope to utilize this information to continue to improve the elective in the upcoming years. An additional limitation of this study is that a formal cost-effective analysis was beyond our scope. However, based on students’ interest in the course and the recognition of benefit, UMMS has continued to provide financial and administrative support for MiM, and the involved physicians (course director, guest speakers) participate on a volunteer basis. Nonetheless, targeting medical schools that serve patient populations where Mandarin speaking immigrants are most highly represented would presumably deliver the highest return on investment in terms of positive impact on patient care.

## Conclusion

Here we have described the implementation and impact of a novel MiM elective developed to address the challenges that LEP Chinese patients encounter in the face of language and cultural barriers. Our course successfully trained students to feel comfortable practicing clinical medicine in Mandarin. We believe that training future physicians to provide care in a patient’s native language represents an effective strategy to mitigate language and cultural barriers in a complementary manner with professional medical interpreters. Future work will seek to more specifically examine the clinical impact of this course.

## Take Home Messages

• A substantial proportion of the US population is not English proficient
• Professional interpreter services are limited and ---utilization is not highly correlated with demand
• Training physicians to provide care in non-English languages could complement interpreters to better patient care
• UMMS successfully implemented a Medicine in Mandarin elective to address both language and cultural barriers to healthcare

## Notes On Contributors

Yingchao Zhong is currently a fellow in the MSTP at the University of Michigan. She completed her BA in Cellular and Molecular Biology and Applied Math at the University of California, Berkeley.

Elaine A. Liu completed her BA in Molecular and Cell Biology at University of California, Berkeley and is currently in the Medical Scientist Training Program at the University of Michigan, completing her MD and PhD in Cellular and Molecular Biology.

Kelly Zhu Young, BA is a current Medical Scientist Training Program (MSTP) student at the University of Michigan. She completed her BA in Neuroscience at Washington University in St. Louis and is currently completing her MD and PhD in Molecular and Integrative Physiology at the University of Michigan.

Jane Miller is a nationally certified healthcare interpreter. She is a full-time interpreter and medical interpreter trainer at Interpreter Services, Michigan Medicine.

Dr. Michael Heung is a Professor of Medicine and nephrologist at the University of Michigan. He serves as the faculty course director for the Medicine in Mandarin pre-clinical elective.
