# Supplementary material for: Medicine in Mandarin: Introducing Native Language Training in a Medical School Curriculum
Source: MedEdPublish (2016). 2019 Nov 15;8:205. [Version 1] doi: 10.15694/mep.2019.000205.1 (PMC10712499; doi:10.15694/mep.2019.000205.1)
Supplement: Peer review history

# Reviewer report: Reviewer response for version 1

**DOI:** 10.21956/mep.19930.r30228

**Published:** 2019-11-24

**Authors:** Leila Niemi-Murola

**Affiliations:** 1 University of Helsinki

This review has been migrated. The reviewer awarded 4 stars out of 5 This is an interesting paper about introducing some native language training in a medical school curriculum. Outside office hours not having a shared language with a patients is sometimes a real challenge for the healthcare personnel. Patient's relatives may be able to tell the history with the physicians and nurses, but it is more difficult to communicate the meaning of results and to negotiate the future plans for the patient.The participants in this course knew some Mandarin Chinese before attending the course and the focus was to teach them the medical vocabulary. There were also sociocultural aspects included in the course curriculum. The students were asked to do a self-assessment before and after the course and there were also weekly tests included in the program. After the course, the students felt much more confident than before attending. It might be that the effect of the course was as much in increasing the participants' cultural competence and self-efficacy beliefs as it was in learning the medical vocabulary. It would be interesting to know if the authors plan to introduce other courses intended to improve the LEP in other most frequently encountered languages.

Reviewer Expertise:

NA

No decision status is available

# Reviewer report: Reviewer response for version 1

**DOI:** 10.21956/mep.19930.r30229

**Published:** 2019-11-19

**Authors:** Sateesh Babu Arja

**Affiliations:** 1 Avalon University School of Medicine

This review has been migrated. The reviewer awarded 4 stars out of 5 A very interesting manuscript. There are many medical schools where English is the medium of instruction, and the local population where the medical school is established may not speak English or May not be proficient in English. It could be a problem for the students if students are doing clerkship in local hospitals in these countries. I like the way authors explained the process for developing a curriculum for the Mandarin language in medical school. These principles can also be applicable to other languages at other schools. And also, teaching methods detailed for each learning session are very interesting. The research methodology is appropriate in this study, and results are adequate, and conclusions drawn based on results are appropriate. Post-course survey results are presented along with the end of course evaluations. It is interesting to see the authors following up with evaluation and research. This manuscript is useful for the curriculum developers and academic administrators who would like to develop language electives in medical school curricula.

Reviewer Expertise:

NA

No decision status is available

# Reviewer report: Reviewer response for version 1

**DOI:** 10.21956/mep.19930.r30230

**Published:** 2019-11-15

**Authors:** Felix Silwimba

**Affiliations:** 1 University of Lusaka

This review has been migrated. The reviewer awarded 5 stars out of 5 this study is motivating for us non.native English language speakers and our patients. it is innovative and valuable lessons have been learnt. it is recommended for all medical educators world over.

Reviewer Expertise:

NA

No decision status is available
